# **Travel distance may have a negative impact on the outcome of deep brain stimulation in Parkinson’s disease**

**DOI:** 10.1038/s41598-025-14519-6

**Published:** 2025-08-16

**Authors:** Hemeda Sahar, Dávid Pintér, Evelyn Pintér, István Balás, József Janszky, Norbert Kovács

**Affiliations:** 1 Department of Neurology, Medical School, University of Pécs, Rét utca 2, Pécs, 7623 Hungary; 2 ELKH-PTE Clinical Neuroscience MR Research Group, Pécs, Hungary; 3 Department of Neurosurgary, Medical School, University of Pécs, Pécs, Hungary

**Keywords:** Parkinson’s disease, Deep brain stimulation, Health-related quality of life, Teleprogramming, Parkinson's disease, Parkinson's disease

## Abstract

Potential beneficial effects of teleprogramming on the efficacy of deep brain stimulation (DBS) have not been studied. We aimed to explore the effects of travel distance on the outcome of DBS in Parkinson’s disease (PD). We retrospectively analyzed travel- and visit-related data of PD patients treated with DBS. Comparing the first-year and five-year outcomes of far-living patients (> 50 km) having the desired number of follow-up visits (*n* = 74) to those of far-living subjects having less visits than optimal (*n* = 54), patients with desired number of visits had better global health-related quality of life according to summary indexes of disease-specific and non-specific scales at five years. Far-living patients with desired number of visits had larger decrease in Parts II, III and IV of the Movement Disorder Society-sponsored Unified Parkinson’s Disease Rating Scale from baseline to the five-year follow-up and more simplified antiparkinsonian medication treatment. Even 40% of PD patients with DBS who live far from the movement disorder center may have less in-person visits than optimal and be on the risk of having worse long-term outcome of DBS than could be achieved. Teleprogramming may help maintaining the long-term efficacy of stimulation in this group of patients.

## Introduction

Introduction of deep brain stimulation (DBS) has been a remarkable milestone in the treatment of several neurological disorders resistant to pharmacotherapy^1–3^. The technical evolution of available systems has resulted in continuously improving efficacy and safety of DBS. Among the released innovations, the NeuroSphere™ platform by Abbott allowing remote programming of the implanted DBS device seems to resolve several difficulties of postoperative care^4–11^. A recent study by our group has shown that among others, teleprogramming can save time and money for both patients and their caregivers, and in line with some other pilot trials, can help the maintenance of DBS care even in special situations such as a pandemic^4–7,12^. In addition, we also found that patients who live further from our center, are of older age or represent higher level of disease severity and disability have less frequent in-person visits at our department both during the first year and five years of stimulation treatment^4–7,12^. However, regular reprogramming of the DBS device that was possible only during in-person visits prior to the introduction of teleprogramming may be necessary especially after the initiation of the stimulation until the optimal settings are found and as the disease progresses to maintain the efficacy of stimulation treatment. Some recent studies suggest that remote programming, even in itself without in-person sessions, may be an effective and safe tool for finding the initial settings, the initial optimalization of the stimulation and maintaining the achieved improvement^13,14^. Patients who cannot have the desired number of in-person visits because of the long-distance, time-consuming, costly, and challenging travels may receive a less efficient treatment that could be achieved which can have serious clinical and economic consequences. To in-depthly investigate this thus far unstudied potential issue of postoperative DBS care, we conducted a single-center, registry-based analysis using data of patients who are being treated with DBS for Parkinson’s disease (PD) in our unit. We hypothesized that far-living patients who had more frequent in-person visits in the first year and during five years of DBS treatment showed a better outcome.

## Results

### Demographics and disease specific data

A total of 161 patients who had at least one-year follow-up and fulfilled all eligibility criteria were identified in our MD registry, of them, 86 subjects had also been followed for at least five years. Of the 75 patients who could not be included in the five-year analyses, 56 patients (74.7%) had a follow-up period shorter than 5 years, 4 patients (5.3%) died before the 5-year visit, and 15 patients (20.0%) were lost to follow up. Of the 15 patients who were lost to follow up, 4 patients lived close (≤ 50 km), while 11 subjects lived far (> 50 km) from our unit.

Most of the included patients (*n* = 128, 79.5%) were living further than 50 km from our MD center meaning their homes were located outside of Baranya county in which Pécs lies. All demographic and disease-specific data of the whole study population and different patient subgroups can be seen in Table 1. One-year and five-year follow-up visits took place between August 9, 2013 and August 12, 2022.

**Table 1:** Characteristics of the study population at deep brain stimulation surgery.

			All patients (*n* = 161)	Subgroup 1^a^ (*n* = 27)	Subgroup 2^a^ (*n* = 6)	Subgroup 3^a^ (*n* = 74)	Subgroup 4^a^ (*n* = 54)	*p*-value^b^
**Demographic** **data**	**Age (years)**		60.6 ± 8.9	60.9 ± 7.8	63.7 ± 7.9	61.0 ± 8.4	59.3 ± 9.9	0.347
	**Sex**	**Males**	110 (68.3)	19 (70.4)	6 (100.0)	48 (64.9)	37 (68.5)	0.578
		**Females**	51 (31.7)	8 (29.6)	0 (0.0)	26 (35.1)	17 (31.5)	
	**Handedness**	**Right**	158 (98.1)	26 (96.3)	6 (100.0)	73 (98.6)	53 (98.2)	0.805
		**Left**	3 (1.9)	1 (3.7)	0 (0.0)	1 (1.4)	1 (1.8)	
	**Education (years)**		13.3 ± 2.9	12.7 ± 3.2	14.2 ± 5.3	13.3 ± 2.6	13.5 ± 3.0	0.890
	**MoCA (version 7)**		24.6 ± 3.3	24.7 ± 3.3	23.9 ± 4.2	24.8 ± 3.1	24.4 ± 3.4	0.624
**Disease-specific** **data**	**Duration of disease (years)**		9.3 ± 4.4	8.8 ± 4.5	9.3 ± 4.7	9.3 ± 4.6	9.4 ± 3.9	0.716
	**Type of disease**	**Tremor-dominant**	36 (22.4)	5 (18.5)	1 (16.7)	17 (23.0)	13 (24.1)	0.483
		**Rigid-akinetic**	73 (45.3)	17 (63.0)	3 (50.0)	32 (43.2)	21 (38.9)	
		**Mixed**	52 (32.3)	5 (18.5)	2 (33.3)	25 (33.8)	20 (37.0)	
	**MDS-UPDRS Part I**		13.6 ± 6.2	15.1 ± 6.6	12.3 ± 5.0	13.4 ± 5.7	13.1 ± 6.8	0.617
	**MDS-UPDRS Part II**		17.3 ± 7.7	19.4 ± 7.7	15.3 ± 3.7	16.4 ± 7.3	17.6 ± 8.3	0.379
	**MDS-UPDRS Part III** ^**c**^		30.4 ± 15.3	34.3 ± 17.1	31.7 ± 9.0	29.9 ± 15.0	30.3 ± 15.1	0.892
	**MDS-UPDRS Part IV**		6.8 ± 4.0	7.7 ± 4.1	6.8 ± 3.3	7.0 ± 4.0	6.2 ± 4.0	0.155
	**EQ-5D-5 L Summary Index**		0.689 ± 0.083	0.709 ± 0.108	0.683 ± 0.075	0.695 ± 0.081	0.684 ± 0.079	0.851
	**PDQ-39 Summary Index**		33.1 ± 14.3	31.8 ± 15.7	33.7 ± 12.6	33.0 ± 19.1	33.4 ± 21.9	0.789
**Antiparkinsonian medication**	**Levodopa treatment**		160 (99.4)	27 (100.0)	5 (83.3)	74 (100.0)	54 (100.0)	NA
	**Dopamine agonist treatment**		89 (55.3)	13 (48.1)	2 (33.3)	48 (64.9)	26 (48.1)	0.059
	**Monoamine oxidase B inhibitor treatment**		33 (20.5)	4 (14.8)	2 (33.3)	20 (27.0)	7 (13.0)	0.054
	**NMDA receptor antagonist treatment**		36 (22.3)	6 (22.2)	1 (16.7)	20 (27.0)	9 (16.7)	0.167
	**COMT inhibitor treatment**		128 (79.5)	20 (74.0)	4 (66.7)	58 (78.4)	46 (85.2)	0.330
	**Anticholinergic treatment**		5 (3.1)	0 (0.0)	1 (16.7)	2 (2.7)	2 (3.7)	0.453
	**Monotherapy**		11 (6.8)	2 (7.4)	0 (0.0)	6 (8.1)	3 (5.6)	0.577
	**Number of different antiparkinsonian drug types**		2.7 ± 0.9	2.4 ± 0.9	3.3 ± 0.8	2.8 ± 0.9	2.5 ± 0.9	0.057
	**Number of different antiparkinsonian drug types**	**0**	0 (0.0)	0 (0.0)	0 (0.0)	0 (0.0)	0 (0.0)	0.350
		**1**	11 (6.8)	2 (7.4)	0 (0.0)	6 (8.1)	3 (5.6)	
		**2**	61 (37.9)	16 (59.3)	1 (16.7)	28 (37.8)	16 (29.6)	
		**3**	60 (37.3)	5 (18.5)	2 (33.3)	27 (36.5)	26 (48.1)	
		**4**	20 (12.4)	3 (11,1)	3 (50.0)	8 (10.8)	6 (11.1)	
		**5**	9 (5.6)	1 (3.7)	0 (0.0)	5 (6.8)	3 (5.6)	
	**Number of tablets (pieces/day)**		7.6 ± 2.7	7.4 ± 2.4	12.3 ± 4.8	7.5 ± 2.3	7.2 ± 2.6	0.304
	**LED for drugs containing levodopa (mg)**		873.3 ± 515.6	857.3 ± 377.0	1833.3 ± 1011.9	858.3 ± 361.1	823.5 ± 532.0	0.265
	**LED for dopamine agonists (mg)**		293.8 ± 234.5	314.3 ± 242.0	235.0 ± 120.2	293.8 ± 215.2	282.9 ± 270.2	0.760
	**Levodopa equivalent daily dose (mg)**		1254.6 ± 586.1	1212.9 ± 443.0	2249.3 ± 1161.4	1265.9 ± 438.6	1184.6 ± 603.7	0.172

Data are mean ± standard deviation or count (%).

^a^Patient subgroups are the followings: Subgroup 1 = patients living close to the center and having the desired in-person visit number; Subgroup 2 = patients living close to the center and having less in-person visits than desired; Subgroup 3 = patients living far from the center and having the desired in-person visit number; and Subgroup 4 = patients living far from the center and having less in-person visits than desired.

^b^Data of patient subgroups 3 and 4 were compared.

^c^MDS-UPDRS Part III was assessed in medication ON state.

COMT = Catechol-O-methyl transferase inhibitor; EQ-5D-5 L = EuroQoL 5-dimension 5-level Health Questionnaire; LED = levodopa equivalent dose; MDS-UPDRS = Movement Disorder Society-sponsored Unified Parkinson’s Disease Rating Scale; MoCA = Montreal Cognitive Assessment; NA = not applicable; NMDA = N-methyl-D- aspartate; PDQ-39 = 39-item Parkinson’s Disease Questionnaire.

### Travel distance and number of in-person visits

The mean travel distance between the homes of the patients and our center was 27.5 ± 14.9 km in the group of patients who were living close to our unit, while it was 220.2 ± 106.7 km in the far-living group. There were negative correlations between travel distance from home to our center and the numbers of in-person visits both during the first-year follow-up period (Spearman’s rho = −0.364, *p* < 0.001) and over five years (Spearman’s rho = −0.368, *p* < 0.001). The portion of patients with five-year follow-up who had the desired number of in-person visits during the first year (at least 3 in-person visits) but visited our unit less frequently than considered as optimal (less than 2 in-person visits per year) from the second year to the five-year follow-up was higher in the far-living group (11/46 vs. 3/19, *p* = 0.041). In the far-living group, patients who visited our unit at least as frequent as considered as optimal had 3.8 ± 1.1 in-person visits in the first year, while 14.5 ± 3.2 visits in the remaining 4 years meaning that these subjects had in general 3.6 in-person visits per year throughout the whole five-year follow-up period. In the group of far-living patients who had less frequent in-person visits than desired, mean visit numbers were 1.8 ± 0.6 and 5.2 ± 0.9 for the first year and the remaining 4 years, respectively, which shows that these patients had a decrease in the number of in-person visits after the first year (generally, 1.8 visits in the first year and 1.3 visits per year in the remaining period of the follow-up). Considering the first-year and five-year data, respectively, 42% (54 subjects out of 128) and 41% (25 subjects out of 61) of the far-living patients had less in-person visits than considered optimal. Travel- and visit-related data can be found in Table 2.

**Table 2:** Travel- and visit-related data.

		All patients	Subgroup 1^a^	Subgroup 2^a^	Subgroup 3^a^	Subgroup 4^a^
**Travel distance between home and center (km)**		180.6 ± 123.3	27.6 ± 14.2	27.0 ± 19.3	210.1 ± 106.9	234.8 ± 106.4
**Number of in-person visits during the first year** ^**b**^		3.2 ± 1.6	5.0 ± 1.3	1.7 ± 0.8	3.8 ± 1.1	1.8 ± 0.6
**Number of in-person visits over five years** ^**c**^		14.3 ± 5.7	19.9 ± 8.7	7.6 ± 2.7	14.5 ± 3.2	7.0 ± 1.5
**Change in patient subgroup status based on change in frequency of in-person visits from one year to five years (number of patients)**	**Change from subgroup 1 to 2**	NA	3	NA	NA	NA
	**Change from subgroup 3 to 4**	NA	NA	NA	19	NA
	**No change**	NA	16	5	34	6
	**Change from subgroup 2 to 1**	NA	NA	1	NA	NA
	**Change from subgroup 4 to 3**	NA	NA	NA	NA	2

Data are mean ± standard deviation or count.

^a^Patient subgroups are the followings: Subgroup 1 = patients living close to the center and having the desired in-person visit number; Subgroup 2 = patients living close to the center and having less in-person visits than desired; Subgroup 3 = patients living far from the center and having the desired in-person visit number; and Subgroup 4 = patients living far from the center and having less in-person visits than desired.

^b^Based on data of the whole study population (*n* = 161) and 27, 6, 74, and 54 patients in subgroups 1, 2, 3, and 4, respectively.

^c^A total of 86 patients had a follow-up period of 5 years, while data of 17, 8, 36, and 25 patients could be analyzed in subgroups 1, 2, 3, and 4, respectively, for the 5-year follow-up.

NA = not applicable.

### Primary endpoint

In the primary outcome (39-item Parkinson’s Disease Questionnaire - PDQ-39 - summary index), there was no significant difference between patients living far from the center and having the desired in-person visit number and living far from the center and having less in-person visits than desired comparing scores assessed at the first-year follow-up visit (considering first-year data of all included patients: 21.8 ± 13.2 vs. 25.3 ± 15.0, *p* = 0.211; analyzing only data of patients who had both first-year and five-year follow-up visits: 19.5 ± 12.8 vs. 20.2 ± 11.4, *p* = 0.813). Of the domains of the PDQ-39, only bodily discomfort showed a significantly larger decrease in patients of subgroup 3 (−17.2 ± 16.7 vs. −7.8 ± 19.2, *p* < 0.001). However, the five-year global global health-related quality of life (HRQoL) was better in patients who had the desired number of in-person visits despite the long distance between their homes and our center (PDQ-39 summary index: 23.3 ± 18.5 vs. 32.2 ± 17.9, *p* = 0.030) and improvement in the PDQ-39 summary index was also higher in this group from baseline (preoperative status) to the five-year follow-up (−9.8 ± 18.7 vs. −1.1 ± 19.8). Significantly lower scores in the mobility, activities of daily living, stigma, and bodily discomfort domains of the PDQ-39 assessed at five years (27.9 ± 25.4 vs. 47.8 ± 29.6, *p* = 0.041; 23.2 ± 20.6 vs. 32.7 ± 17.2, *p* = 0.030; 17.8 ± 19.8 vs. 36.2 ± 20.8, *p* = 0.001; and 23.8 ± 16.7 vs. 34.1 ± 18.5, *p* = 0.036, respectively) might have contributed to the better outcome in the group of patients who visited our unit as frequently as it considered as optimal by our protocol. Improvement in the mobility, activities of daily living, stigma, communication, and bodily discomfort domains of the PDQ-39 from baseline to the five-year follow-up was larger in subgroup 3 (Table 3).

**Table 3:** The primary outcomes at the first-year and five-year follow-up visits in patient groups 3 and 4.

		Preoperative^a^		*p*-value	First year follow-up^a^		*p*-value	Five-year follow-up^b^		*p*-value
		Subgroup 3	Subgroup 4		Subgroup 3	Subgroup 4		Subgroup 3	Subgroup 4	
**PDQ-39 Summary Index**		33.0 ± 19.1	33.4 ± 21.9	0.789	21.8 ± 13.2	25.3 ± 15.0	0.211	23.3 ± 18.5	32.2 ± 17.9	0.030
**Change in PDQ-39 Summary Index** ^**c**^		NA	NA	NA	−11.3 ± 16.0	−8.2 ± 18.8	0.217	−9.8 ± 18.7	−1.1 ± 19.8	0.049
**PDQ-39 Mobility**		37.2 ± 23.2	33.6 ± 25.7	0.446	23.8 ± 17.7	29.5 ± 22.5	0.449	27.9 ± 25.4	47.8 ± 29.6	0.041
**Change in PDQ-39 Mobility** ^**c**^		NA	NA	NA	−13.3 ± 20.2	−4.1 ± 24.3	0.075	−9.2 ± 24.0	14.1 ± 27.5	0.017
**PDQ39 Activities of daily living**		32.8 ± 18.0	30.4 ± 19.8	0.423	18.8 ± 12.5	20.5 ± 15.0	0.687	23.2 ± 20.6	32.7 ± 17.2	0.030
**Change in PDQ39 Activities of daily living** ^**c**^		NA	NA	NA	−13.8 ± 15.6	−10.0 ± 17.6	0.112	−9.7 ± 19.2	2.3 ± 18.4	0.047
**PDQ-39 Emotional well-being**		35.4 ± 22.5	37.7 ± 24.0	0.647	20.8 ± 16.6	25.4 ± 18.1	0.289	24.8 ± 22.2	29.9 ± 18.2	0.119
**Change in PDQ-39 Emotional well-being** ^**c**^		NA	NA	NA	−14.4 ± 19.4	−9.9 ± 20.4	0.344	−10.5 ± 22.4	−7.7 ± 21.8	0.630
**PDQ-39 Stigma**		39.0 ± 23.1	39.1 ± 27.4	0.791	15.5 ± 14.7	20.5 ± 20.1	0.289	17.8 ± 19.8	36.2 ± 20.8	0.001
**Change in PDQ-39 Stigma** ^**c**^		NA	NA	NA	−23.3 ± 19.0	−18.5 ± 23.7	0.175	−21.0 ± 21.5	−3.0 ± 23.9	0.029
**PDQ-39 Social support**		23.5 ± 15.6	26.3 ± 16.8	0.654	22.3 ± 10.9	25.5 ± 11.4	0.389	16.2 ± 16.9	17.6 ± 11.4	0.258
**Change in PDQ-39 Social support** ^**c**^		NA	NA	NA	−1.2 ± 12.5	−0.8 ± 13.8	0.084	−7.4 ± 16.3	−8.6 ± 14.4	0.149
**PDQ-39 Cognition**		29.5 ± 16.5	29.6 ± 15.6	0.938	28.7 ± 11.2	28.7 ± 10.0	0.213	24.1 ± 14.1	24.5 ± 13.4	0.595
**Change in PDQ-39 Cognition** ^**c**^		NA	NA	NA	−0.8 ± 13.9	−1.0 ± 13.1	0.589	−5.5 ± 15.8	−5.1 ± 14.5	0.136
**PDQ-39 Communication**		31.2 ± 16.1	33.6 ± 22.1	0.395	22.8 ± 15.8	21.6 ± 14.6	0.969	24.6 ± 18.1	32.9 ± 17.8	0.081
**Change in PDQ-39 Communication** ^**c**^		NA	NA	NA	−9.4 ± 15.9	−11.9 ± 18.9	0.301	− 6.6 ± 17.3	−0.8 ± 16.2	< 0.001
**PDQ-39 Bodily discomfort**		39.3 ± 22.8	36.3 ± 25.3	0.421	22.0 ± 11.1	28.4 ± 11.9	0.098	23.8 ± 16.7	34.1 ± 18.5	0.036
**Change in PDQ-39 Bodily discomfort** ^**c**^		NA	NA	NA	−17.2 ± 16.7	−7.8 ± 19.2	< 0.001	−15.4 ± 19.7	−2.2 ± 21.9	< 0.001
**EQ-5D-5 L Summary Index**		0.695 ± 0.081	0.684 ± 0.079	0.851	0.813 ± 0.112	0.755 ± 0.143	0.231	0.802 ± 0.122	0.705 ± 0.181	0.033
**Change in EQ-5D-5 L Summary Index** ^**c**^		NA	NA	NA	−0.118 ± 0.098	−0.071 ± 0.103	0.009	−0.107 ± 0.101	−0.022 ± 0.132	< 0.001
**EQ-5D-5 L VAS**		63.7 ± 15.5	61.8 ± 20.0	0.443	70.7 ± 13.4	67.6 ± 19.0	0.732	65.6 ± 17.8	57.8 ± 18.2	0.106
**EQ-5D-5 L Mobility**	**No problems**	2 (2.7)	3 (5.6)	0.376	15 (20.3)	4 (7.4)	0.203	6 (16.7)	1 (4.0)	0.009
	**Slight problems**	19 (25.7)	18 (33.3)		25 (33.8)	22 (40.7)		15 (41.7)	6 (24.0)	
	**Moderate problems**	30 (40.5)	20 (37.0)		32 (43.2)	25 (46.3)		14 (38.8)	9 (36.0)	
	**Severe problems**	23 (31.1)	12 (22.2)		2 (2.7)	3 (5.6)		1 (2.8)	8 (32.0)	
	**Unable to walk about**	0 (0.0)	1 (1.9)		0 (0.0)	0 (0.0)		0 (0.0)	1 (4.0)	
**EQ-5D-5 L Self-care**	**No problems**	4 (5.4)	3 (5.6)	0.623	12 (16.2)	10 (18.6)	0.080	5 (13.9)	2 (8.0)	0.202
	**Slight problems**	28 (37.8)	16 (29.6)		46 (62.2)	22 (40.7)		18 (50.0)	7 (28.0)	
	**Moderate problems**	32 (43.2)	28 (51.8)		14 (18.9)	20 (37.0)		10 (27.8)	13 (52.0)	
	**Severe problems**	10 (13.6)	6 (11.1)		2 (2.7)	2 (3.7)		3 (8.3)	4 (16.0)	
	**Unable to wash or dress**	0 (0.0)	1 (1.9)		0 (0.0)	0 (0.0)		0 (0.0)	0 (0.0)	
**EQ-5D-5 L** **Usual activities**	**No problems**	3 (4.1)	2 (3.7)	0.578	16 (21.6)	7 (13.0)	0.155	6 (16.7)	2 (8.0)	0.023
	**Slight problems**	22 (29.7)	16 (29.6)		35 (47.3)	19 (35.1)		17 (47.2)	4 (16.0)	
	**Moderate problems**	41 (55.4)	25 (46.3)		20 (27.0)	24 (44.4)		12 (33.3)	14 (56.0)	
	**Severe problems**	8 (10.8)	10 (18.5)		3 (4.1)	3 (5.6)		1 (2.8)	4 (16.0)	
	**Unable to do usual activities**	0 (0.0)	1 (1.9)		0 (0.0)	1 (1.9)		0 (0.0)	1 (1.9)	
**EQ-5D-5 L Pain/** **discomfort**	**No pain or discomfort**	3 (4.1)	2 (3.7)	0.088	18 (24.3)	8 (14.8)	0.290	8 (22.2)	2 (8.0)	0.185
	**Slight pain or discomfort**	15 (20.3)	13 (24.1)		30 (40.6)	18 (33.3)		13 (36.1)	6 (24.0)	
	**Moderate pain or discomfort**	49 (66.2)	29 (53.6)		20 (27.0)	21 (38.9)		12 (33.3)	10 (40.0)	
	**Severe pain or discomfort**	6 (8.1)	5 (9.3)		5 (6.8)	4 (7.4)		2 (11.1)	5 (20.0)	
	**Extreme pain or discomfort**	1 (1.3)	5 (9.3)		1 (1.3)	3 (5.6)		1 (2.7)	2 (8.0)	
**EQ-5D-5 L Anxiety/** **depression**	**Not anxious or depressed**	34 (45.9)	27 (50.0)	0.280	39 (52.7)	31 (57.4)	0.860	16 (44.4)	5 (20.0)	0.086
	**Slightly anxious or depressed**	22 (29.7)	19 (35.2)		25 (33.8)	16 (29.6)		11 (30.6)	6 (24.0)	
	**Moderately anxious or depressed**	18 (24.4)	7 (12.9)		10 (13.5)	7 (13.0)		8 (22.2)	12 (48.0)	
	**Severely anxious or depressed**	0 (0.0)	1 (1.9)		0 (0.0)	0 (0.0)		1 (2.8)	2 (8.0)	
	**Extremely anxious or depressed**	0 (0.0)	0 (0.0)		0 (0.0)	0 (0.0)		0 (0.0)	0 (0.0)	
**SE-ADL**	**100%**	0 (0.0)	0 (0.0)	0.574	6 (8.1)	2 (3.7)	0.536	2 (5.6)	1 (4.0)	0.021
	**90%**	20 (27.0)	15 (27.7)		24 (32.4)	17 (31.5)		12 (33.3)	3 (12.0)	
	**80%**	27 (36.5)	14 (26.0)		23 (31.2)	13 (24.1)		8 (22.1)	3 (12.0)	
	**70%**	12 (16.1)	16 (29.5)		13 (17.6)	15 (27.7)		6 (16.7)	1 (4.0)	
	**60%**	8 (10.8)	6 (11.1)		7 (9.5)	7 (13.0)		6 (16.7)	4 (16.0)	
	**50%**	2 (2.7)	0 (0.0)		0 (0.0)	0 (0.0)		1 (2.8)	6 (24.0)	
	**40%**	3 (4.1)	1 (1.9)		0 (0.0)	0 (0.0)		0 (0.0)	3 (12.0)	
	**30%**	1 (1.4)	1 (1.9)		1 (1.4)	0 (0.0)		1 (2.8)	2 (8.0)	
	**20%**	1 (1.4)	1 (1.9)		0 (0.0)	0 (0.0)		0 (0.0)	1 (4.0)	
	**10%**	0 (0.0)	0 (0.0)		0 (0.0)	0 (0.0)		0 (0.0)	1 (4.0)	
	**0%**	0 (0.0)	0 (0.0)		0 (0.0)	0 (0.0)		0 (0.0)	0 (0.0)	

Data are mean ± standard deviation or count (%).

Patient subgroups are the followings: Subgroup 3 = patients living far from the center and having the desired in-person visit number; while Subgroup 4 = patients living far from the center and having less in-person visits than desired.

^a^Based on data of 74 patients in subgroup 3 and 54 subjects in subgroup 4.

^b^Based on data of 36 patients in subgroup 3 and 25 subjects in subgroup 4.

^c^Change from preoperative status to first-year and five-year status.

EQ-5D-5 L = EuroQoL 5-dimension 5-level Health Questionnaire; EQ-5D-5 L VAS = Visual Analogue Scale of the EuroQoL 5-dimension 5-level Health Questionnaire; PDQ-39 = 39-item Parkinson’s Disease Questionnaire, SE-ADL = Schwab and England Activities of Daily Living Scale.

The findings on HRQoL were also confirmed by the five-year summary index of the EuroQoL 5-Dimension 5-Level (EQ-5D-5 L, 0.802 ± 0.122 vs. 0.705 ± 0.181, *p* = 0.033). Improvement in the EQ-5D-5 L summary index from baseline to the five-year follow-up was significantly larger in far-living patients with regular in-person visits both after one year of stimulation and at five years (−0.118 ± 0.098 vs. −0.071 ± 0.103; *p* = 0.009 and − 0.107 ± 0.101 vs. −0.022 ± 0.132; *p* < 0.001, respectively). Of the dimensions of the EQ-5D-5 L, numbers of patients who reported better outcome on mobility and usual activities were significantly higher in group of patients having the desired number of in-person visits over five years of stimulation (*p* = 0.009 and *p* = 0.023, respectively). The five-year Shwab-England Activities of Daily Living Scale (SE-ADL) outcome was also better in patients of subgroup 3 (*p* = 0.021, Table 3).

Comparing data of subgroups 1 and 3, we found no significant difference in HRQoL measures **(**Table 4**).**

**Table 4:** The primary outcomes at the first-year and five-year follow-up visits in patient groups 1 and 3.

		Preoperative^a^		*p*-value	First year follow-up^a^		*p*-value	Five-year follow-up^b^		*p*-value
		Subgroup 1	Subgroup 3		Subgroup 1	Subgroup 3		Subgroup 1	Subgroup 3	
**PDQ-39 Summary Index**		33.1 ± 14.3	33.0 ± 19.1	0.928	22.6 ± 17.9	21.8 ± 13.2	0.803	22.3 ± 17.8	23.3 ± 18.5	0.889
**Change in PDQ-39 Summary Index** ^**c**^		NA	NA	NA	− 10.6 ± 12.6	−11.3 ± 16.0	0.855	−10.7 ± 17.7	−9.8 ± 18.7	0.819
**PDQ-39 Mobility**		35.6 ± 24.2	37.2 ± 23.2	0.795	26.4 ± 18.4	23.8 ± 17.7	0.782	25.9 ± 20.1	27.9 ± 25.4	0.786
**Change in PDQ-39 Mobility** ^**c**^		NA	NA	NA	−9.3.3 ± 24.5	−13.3 ± 20.2	0.208	−9.8 ± 18.6	−9.2 ± 24.0	0.689
**PDQ39 Activities of daily living**		28.4 ± 23.5	32.8 ± 18.0	0.388	19.2 ± 11.1	18.8 ± 12.5	0.788	22.3 ± 19.7	23.2 ± 20.6	0.901
**Change in PDQ39 Activities of daily living** ^**c**^		NA	NA	NA	−9.3 ± 13.4	−13.8 ± 15.6	0.234	−6.1 ± 16.7	−9.7 ± 19.2	0.199
**PDQ-39 Emotional well-being**		28.9 ± 21.9	35.4 ± 22.5	0.297	18.5 ± 13.2	20.8 ± 16.6	0.301	19.4 ± 16.2	24.8 ± 22.2	0.321
**Change in PDQ-39 Emotional well-being** ^**c**^		NA	NA	NA	−10.4 ± 15.6	−14.4 ± 19.4	0.256	−9.5 ± 17.8	−10.5 ± 22.4	0.677
**PDQ-39 Stigma**		30.8 ± 22.4	39.0 ± 23.1	0.234	11.1 ± 12.4	15.5 ± 14.7	0.294	14.1 ± 12.5	17.8 ± 19.8	0.601
**Change in PDQ-39 Stigma** ^**c**^		NA	NA	NA	−19.7 ± 15.7	−23.3 ± 19.0	0.336	−16.7 ± 15.9	−21.0 ± 21.5	0.652
**PDQ-39 Social support**		25.5 ± 21.4	23.5 ± 15.6	0.659	23.9 ± 9.8	22.3 ± 10.9	0.854	17.7 ± 13.8	16.2 ± 16.9	0.789
**Change in PDQ-39 Social support** ^**c**^		NA	NA	NA	−1.3 ± 10.8	−1.2 ± 12.5	0.903	−7.8 ± 14.9	−7.4 ± 16.3	0.825
**PDQ-39 Cognition**		30.3 ± 17.5	29.5 ± 16.5	0.853	27.4 ± 10.1	28.7 ± 11.2	0.788	25.6 ± 18.9	24.1 ± 14.1	0.703
**Change in PDQ-39 Cognition** ^**c**^		NA	NA	NA	−2.8 ± 11.7	−0.8 ± 13.9	0.120	−4.7 ± 11.9	−5.5 ± 15.8	0.715
**PDQ-39 Communication**		30.4 ± 10.2	31.2 ± 16.1	0.811	20.3 ± 13.4	22.8 ± 15.8	0.723	23.8 ± 17.6	24.6 ± 18.1	0.869
**Change in PDQ-39 Communication** ^**c**^		NA	NA	NA	−10.1 ± 11.8	−9.4 ± 15.9	0.866	− 6.6 ± 15.9	− 6.6 ± 17.3	0.922
**PDQ-39 Bodily discomfort**		38.6 ± 26.9	39.3 ± 22.8	0.913	20.9 ± 10.0	22.0 ± 11.1	0.899	22.1 ± 18.3	23.8 ± 16.7	0.826
**Change in PDQ-39 Bodily discomfort** ^**c**^		NA	NA	NA	−17.8 ± 13.3	−17.2 ± 16.7	0.922	−16.5 ± 20.4	−15.4 ± 19.7	0.801
**EQ-5D-5 L Summary Index**		0.709 ± 0.108	0.695 ± 0.081	0.885	0.822 ± 0.121	0.813 ± 0.112	0.944	0.820 ± 0.139	0.802 ± 0.122	0.817
**Change in EQ-5D-5 L Summary Index** ^**c**^		NA	NA	NA	0.113 ± 0.086	0.118 ± 0.098	0.931	0.111 ± 0.112	0.107 ± 0.101	0.899
**EQ-5D-5 L VAS**		63.1 ± 14.2	63.7 ± 15.5	0.911	73.8 ± 11.7	70.7 ± 13.4	0.689	68.4 ± 14.5	65.6 ± 17.8	0.810
**EQ-5D-5 L Mobility**	**No problems**	1 (3.7)	2 (2.7)	0.401	5 (18.5)	15 (20.3)	0.543	3 (17.6)	6 (16.7)	0.711
	**Slight problems**	6 (22.2)	19 (25.7)		9 (33.3)	25 (33.8)		6 (35.3)	15 (41.7)	
	**Moderate problems**	10 (37.0)	30 (40.5)		11 (40.7)	32 (43.2)		8 (47.1)	14 (38.8)	
	**Severe problems**	10 (37.0)	23 (31.1)		2 (7.4)	2 (2.7)		0 (0.0)	1 (2.8)	
	**Unable to walk about**	0 (0.0)	0 (0.0)		0 (0.0)	0 (0.0)		0 (0.0)	0 (0.0)	
**EQ-5D-5 L Self-care**	**No problems**	2 (7.4)	4 (5.4)	0.655	4 (14.8)	12 (16.2)	0.599	3 (17.6)	5 (13.9)	0.765
	**Slight problems**	10 (37.0)	28 (37.8)		16 (59.3)	46 (62.2)		9 (52.9)	18 (50.0)	
	**Moderate problems**	11 (40.1)	32 (43.2)		6 (22.2)	14 (18.9)		4 (23.5)	10 (27.8)	
	**Severe problems**	4 (14.8)	10 (13.6)		1 (3.7)	2 (2.7)		1 (5.9)	3 (8.3)	
	**Unable to wash or dress**	0 (0.0)	0 (0.0)		0 (0.0)	0 (0.0)		0 (0.0)	0 (0.0)	
**EQ-5D-5 L** **Usual activities**	**No problems**	3 (11.1)	3 (4.1)	0.512	7 (25.9)	16 (21.6)	0.339	3 (17.6)	6 (16.7)	0.532
	**Slight problems**	8 (29.7)	22 (29.7)		13 (48.1)	35 (47.3)		10 (58.8)	17 (47.2)	
	**Moderate problems**	12 (44.4)	41 (55.4)		5 (18.5)	20 (27.0)		3 (17.6)	12 (33.3)	
	**Severe problems**	4 (14.8)	8 (10.8)		2 (7.4)	3 (4.1)		1 (5.9)	1 (2.8)	
	**Unable to do usual activities**	0 (0.0)	0 (0.0)		0 (0.0)	0 (0.0)		0 (0.0)	0 (0.0)	
**EQ-5D-5 L Pain/** **discomfort**	**No pain or discomfort**	2 (7.4)	3 (4.1)	0.189	6 (22.2)	18 (24.3)	0.476	4 (23.5)	8 (22.2)	0.817
	**Slight pain or discomfort**	6 (22.2)	15 (20.3)		11 (40.7)	30 (40.6)		6 (35.3)	13 (36.1)	
	**Moderate pain or discomfort**	15 (55.6)	49 (66.2)		6 (22.2)	20 (27.0)		6 (35.3)	12 (33.3)	
	**Severe pain or discomfort**	4 (14.8)	6 (8.1)		4 (14.8)	5 (6.8)		1 (5.9)	2 (11.1)	
	**Extreme pain or discomfort**	0 (0.0)	1 (1.3)		0 (0.0)	1 (1.3)		0 (0.0)	1 (2.7)	
**EQ-5D-5 L Anxiety/** **depression**	**Not anxious or depressed**	13 (48.1)	34 (45.9)	0.789	13 (48.1)	39 (52.7)	0.701	7 (41.2)	16 (44.4)	0.765
	**Slightly anxious or depressed**	7 (26.0)	22 (29.7)		9 (33.3)	25 (33.8)		6 (35.3)	11 (30.6)	
	**Moderately anxious or depressed**	7 (26.0)	18 (24.4)		5 (18.5)	10 (13.5)		3 (17.6)	8 (22.2)	
	**Severely anxious or depressed**	0 (0.0)	0 (0.0)		0 (0.0)	0 (0.0)		1 (5.9)	1 (2.8)	
	**Extremely anxious or depressed**	0 (0.0)	0 (0.0)		0 (0.0)	0 (0.0)		0 (0.0)	0 (0.0)	
**SE-ADL**	**100%**	1 (3.7)	0 (0.0)	0.478	2 (7.4)	6 (8.1)	0.501	1 (5.8)	2 (5.6)	0.389
	**90%**	6 (22.2)	20 (27.0)		10 (37.0)	24 (32.4)		5 (29.4)	12 (33.3)	
	**80%**	8 (29.6)	27 (36.5)		10 (37.0)	23 (31.2)		5 (29.4)	8 (22.1)	
	**70%**	3 (11.1)	12 (16.1)		3 (11.1)	13 (17.6)		4 (23.5)	6 (16.7)	
	**60%**	3 (11.1)	8 (10.8)		1 (3.7)	7 (9.5)		1 (5.9)	6 (16.7)	
	**50%**	2 (7.4)	2 (2.7)		0 (0.0)	0 (0.0)		0 (0.0)	1 (2.8)	
	**40%**	1 (3.7)	3 (4.1)		1 (3.7)	0 (0.0)		1 (5.9)	0 (0.0)	
	**30%**	2 (7.4)	1 (1.4)		0 (0.0)	1 (1.4)		0 (0.0)	1 (2.8)	
	**20%**	1 (3.7)	1 (1.4)		0 (0.0)	0 (0.0)		0 (0.0)	0 (0.0)	
	**10%**	0 (0.0)	0 (0.0)		0 (0.0)	0 (0.0)		0 (0.0)	0 (0.0)	
	**0%**	0 (0.0)	0 (0.0)		0 (0.0)	0 (0.0)		0 (0.0)	0 (0.0)	

Data are mean ± standard deviation or count (%).

Patient subgroups are the followings: Subgroup 1 = patients living close to the center and having the desired in-person visit number; while Subgroup 3 = patients living far from the center and having the desired in-person visit number.

^a^Based on data of 27 patients in subgroup 1 and 74 subjects in subgroup 3.

^b^Based on data of 17 patients in subgroup 1 and 36 subjects in subgroup 3.

^c^Change from preoperative status to first-year and five-year status.

EQ-5D-5 L = EuroQoL 5-dimension 5-level Health Questionnaire; EQ-5D-5 L VAS = Visual Analogue Scale of the EuroQoL 5-dimension 5-level Health Questionnaire; PDQ-39 = 39-item Parkinson’s Disease Questionnaire, SE-ADL = Schwab and England Activities of Daily Living Scale.

### Secondary endpoint

On the first-year follow-up visit, the motor and non-motor symptomatic control did not differ significantly between subgroups 3 and 4. However, at five years, far-living patients with the desired number of in-person visits reported a better outcome on the motor experiences of daily living (Movement Disorder Society-Sponsored Unified Parkinson’s Disease Rating Scale - MDS-UPDRS - Part II: 14.7 ± 8.7 points vs. 18.7 ± 8.3 points, *p* = 0.035). In this group of patients, compared to baseline, the improvement in MDS-UPDRS Parts II, III and IV was significantly higher (−1.7 ± 7.9 points vs. 1.0 ± 8.3 points, *p* < 0.001; −8.5 ± 13.5 points vs. −3.5 ± 14.8 points, *p* = 0.012; and − 2.5 ± 3.6 points vs. −0.8 ± 3.9 points, *p* = 0.049, respectively, Table 5).

**Table 5:** The secondary outcomes at the first-year and five-year follow-up visits in patient groups 3 and 4.

		Preoperative^a^		*p*-value	First year follow-up^a^		*p*-value	Five-year follow-up^b^		*p*-value
		Subgroup 3	Subgroup 4		Subgroup 3	Subgroup 4		Subgroup 3	Subgroup 4	
**MDS-UPDRS Part I**		13.4 ± 5.7	13.1 ± 6.8	0.617	10.2 ± 5.0	10.3 ± 6.5	0.966	10.6 ± 6.2	10.6 ± 7.0	0.770
**Change in MDS-UPDRS Part I** ^**c**^		NA	NA	NA	− 3.1 ± 5.3	−2.8 ± 6.6	0.782	−2.7 ± 6.0	−2.5 ± 6.9	0.685
**MDS-UPDRS Part II**		16.4 ± 7.3	17.6 ± 8.3	0.379	11.4 ± 6.7	13.4 ± 7.8	0.177	14.7 ± 8.7	18.7 ± 8.3	0.035
**Change in MDS-UPDRS Part II** ^**c**^		NA	NA	NA	− 4.9 ± 6.9	−4.2 ± 8.1	0.836	−1.7 ± 7.9	1.0 ± 8.3	< 0.001
**MDS-UPDRS Part III** ^**d**^		29.9 ± 15.0	30.3 ± 15.1	0.892	20.7 ± 13.1	21.6 ± 12.0	0.369	21.4 ± 12.5	26.8 ± 14.4	0.070
**Change in MDS-UPDRS Part III** ^**c**^		NA	NA	NA	− 9.2 ± 14.0	−8.8 ± 13.7	0.600	−8.5 ± 13.5	−3.5 ± 14.8	0.012
**MDS-UPDRS Part IV**		7.0 ± 4.0	6.3 ± 4.0	0.235	3.5 ± 3.4	2.9 ± 3.4	0.193	4.4 ± 3.4	5.6 ± 3.6	0.255
**Change in MDS-UPDRS Part IV** ^**c**^		NA	NA	NA	− 3.5 ± 3.7	−3.5 ± 3.8	0.989	−2.5 ± 3.6	−0.8 ± 3.9	0.049
**Levodopa treatment**		74 (100.0)	54 (100.0)	NA	49 (66.2)	36 (66.7)	0.990	20 (55.6)	22 (88.0)	0.007
**Dopamine agonist treatment**		48 (64.9)	22 (40.7)	0.059	38 (51.3)	24 (44.4)	0.191	32 (88.9)	14 (56.0)	0.003
**Monoamine oxidase B inhibitor treatment**		20 (12.4)	7 (13.0)	0.054	4 (5.4)	1 (1.9)	0.471	5 (13.8)	9 (36.0)	0.043
**NMDA receptor antagonist treatment**		20 (27.0)	9 (16.7)	0.167	5 (6.8)	3 (5.6)	0.767	4 (11.1)	2 (8.0)	0.688
**COMT inhibitor treatment**		58 (78.4)	46 (85.2)	0.330	43 (58.1)	36 (66.7)	0.372	25 (69.4)	19 (76.0)	0.574
**Anticholinergic treatment**		2 (2.7)	2 (3.7)	0.453	0 (0.0)	0 (0.0)	NA	1 (2.8)	1 (4.0)	0.782
**Monotherapy**		6 (8.1)	3 (5.6)	0.577	34 (45.9)	28 (51.6)	0.556	9 (25.0)	3 (12.0)	0.089
**Number of different antiparkinsonian drug types**		2.8 ± 0.9	2.5 ± 0.9	0.057	1.6 ± 0.6	1.6 ± 0.7	0.925	1.9 ± 0.8	2.0 ± 0.9	0.269
**Change in number of different antiparkinsonian drug types** ^**c**^		NA	NA	NA	−1.2 ± 0.9	−0.9 ± 1.0	0.055	−1.0 ± 0.8	−0.5 ± 0.9	0.066
**Number of different antiparkinsonian drug types**	**0**	0 (0.0)	0 (0.0)	0.350	0 (0.0)	0 (0.0)	0.166	1 (2.8)	0 (0.0)	0.253
	**1**	6 (8.1)	3 (5.6)		34 (45.9)	29 (53.7)		9 (25.0)	3 (12.0)	
	**2**	28 (37.8)	16 (29.6)		33 (44.6)	17 (31.5)		19 (52.8)	12 (48.0)	
	**3**	27 (36.5)	26 (48.1)		7 (9.5)	8 (14.8)		5 (13.9)	4 (16.0)	
	**4**	8 (10.8)	6 (11.1)		0 (0.0)	0 (0.0)		2 (5.6)	4 (16.0)	
	**5**	5 (6.8)	3 (5.6)		0 (0.0)	0 (0.0)		0 (0.0)	2 (8.0)	
**Number of tablets (pieces/day)**		7.5 ± 2.3	7.2 ± 2.6	0.304	4.5 ± 2.5	4.8 ± 3.0	0.562	5.6 ± 2.7	6.6 ± 3.0	0.355
**Change in number of tablets (pieces/day)** ^**c**^		NA	NA	NA	−3.0 ± 2.7	−2.4 ± 3.2	0.107	−1.9 ± 2.5	−0.6 ± 2.9.	0.039
**LED for drugs containing levodopa (mg)**		858.3 ± 361.1	823.5 ± 532.0	0.265	384.8 ± 414.4	388.2 ± 368.4	0.767	401.1 ± 370.5	400.3 ± 363.8	0.559
**Change in LED for drugs containing levodopa (mg)** ^**c**^		NA	NA	NA	−497.1 ± 335.3	−405.7 ± 484.9	0.150	−455.8 ± 399.8	−421.8 ± 496.9	0.991
**LED for dopamine agonists (mg)**		293.8 ± 215.2	282.9 ± 270.2	0.760	248.4 ± 265.5	228.8 ± 215.8	0.980	212.9 ± 206.7	218.5 ± 264.8	0.605
**Change in LED for dopamine agonists (mg)** ^**c**^		NA	NA	NA	−49.6 ± 258.9	−55.7 ± 215.4	0.732	−55.4 ± 210.8	−60.7 ± 269.1	0.788
**Levodopa equivalent daily dose (mg)**		1265.9 ± 438.6	1184.6 ± 603.7	0.172	650.1 ± 461.6	653.0 ± 429.9	0.859	671.6 ± 391.3	701.5 ± 403.2	0.700
**Change in levodopa equivalent daily dose (mg)** ^**c**^		NA	NA	NA	−612.9 ± 443.6	−514.7 ± 511.4	0.389	−565.0 ± 405.4	−480.7 ± 489.8	0.298

Data are mean ± standard deviation or count (%).

Patient subgroups are the followings: Subgroup 3 = patients living far from the center and having the desired in-person visit number; while Subgroup 4 = patients living far from the center and having less in-person visits than desired.

^a^Based on data of 74 patients in subgroup 3 and 54 subjects in subgroup 4.

^b^Based on data of 36 patients in subgroup 3 and 25 subjects in subgroup 4.

^c^Change from preoperative status to first-year and five-year status.

^d^Preoperatively, MDS-UPDRS Part III was assessed in medication ON state, while it was assessed in medication ON and stimulation ON state at follow-up visits.

COMT = Catechol-O-methyl transferase inhibitor; LED = levodopa equivalent dose; MDS-UPDRS = Movement Disorder Society-sponsored Unified Parkinson’s Disease Rating Scale; NA = not applicable; NMDA = N-methyl-D- aspartate.

At five years, the portions of patients on levodopa and monoamine oxidase B inhibitor treatment were higher in the far-living group of patients who visited our unit less frequently as considered as optimal, while portion of patients on dopamine agonist treatment was higher in the far-living patient group with the desired number of in-person visits over five years. There was a higher decrease in the number of patients on monotherapy from the one-year visit to the five-year follow up. The number of different antiparkinsonian medication types could be decreased in a tendentiously higher manner in far-living patients with the desired number of in-person visits and manner of decrease in the number of tablets daily taken from baseline to five years was also significantly higher in this group (−1.9 ± 2.5 vs. −0.6 ± 2.9, *p* = 0.039; Table 5).

Comparing data of subgroups 1 and 3, we found no significant differences in motor and non-motor symptomatic control and per oral antiparkinsonian treatment (Table 6).

**Table 6:** The secondary outcomes at the first-year and five-year follow-up visits in patient groups 1 and 3.

		Preoperative^a^		*p*-value	First year follow-up^a^		*p*-value	Five-year follow-up^b^		*p*-value
		Subgroup 1	Subgroup 3		Subgroup 1	Subgroup 3		Subgroup 1	Subgroup 3	
**MDS-UPDRS Part I**		15.1 ± 6.6	13.4 ± 5.7	0.445	10.6 ± 4.3	10.2 ± 5.0	0.901	11.2 ± 5.9	10.6 ± 6.2	0.799
**Change in MDS-UPDRS Part I** ^**c**^		NA	NA	NA	− 4.5 ± 5.1	− 3.1 ± 5.3	0.305	−3.8 ± 5.4	−2.7 ± 6.0	0.098
**MDS-UPDRS Part II**		19.4 ± 7.7	16.4 ± 7.3	0.202	13.1 ± 7.3	11.4 ± 6.7	0.183	17.4 ± 10.9	14.7 ± 8.7	0.086
**Change in MDS-UPDRS Part II** ^**c**^		NA	NA	NA	− 6.3 ± 6.1	− 4.9 ± 6.9	0.085	−2.0 ± 6.6	−1.7 ± 7.9	0.101
**MDS-UPDRS Part III** ^**d**^		34.3 ± 17.1	29.9 ± 15.0	0.706	22.4 ± 16.5	20.7 ± 13.1	0.154	23.8 ± 14.7	21.4 ± 12.5	0.206
**Change in MDS-UPDRS Part III** ^**c**^		NA	NA	NA	− 11.8 ± 15.6	− 9.2 ± 14.0	0.091	−10.5 ± 15.1	−8.5 ± 13.5	0.121
**MDS-UPDRS Part IV**		7.7 ± 4.1	7.0 ± 4.0	0.211	3.3 ± 3.1	3.5 ± 3.4	0.511	4.7 ± 4.4	4.4 ± 3.4	0.775
**Change in MDS-UPDRS Part IV** ^**c**^		NA	NA	NA	− 4.4 ± 3.9	− 3.5 ± 3.7	0.243	−3.0 ± 3.9	−2.5 ± 3.6	0.233
**Levodopa treatment**		27 (100.0)	74 (100.0)	NA	16 (59.2)	49 (66.2)	0.445	7 (41.2)	20 (55.6)	0.344
**Dopamine agonist treatment**		13 (48.1)	48 (64.9)	0.098	17 (63.0)	38 (51.3)	0.103	14 (82.3)	32 (88.9)	0.778
**Monoamine oxidase B inhibitor treatment**		4 (14.8)	20 (12.4)	0.754	2 (7.4)	4 (5.4)	0.412	2 (11.8)	5 (13.8)	0.799
**NMDA receptor antagonist treatment**		6 (22.2)	20 (27.0)	0.467	2 (7.4)	5 (6.8)	0.759	2 (11.8)	4 (11.1)	0.903
**COMT inhibitor treatment**		20 (74.0)	58 (78.4)	0.530	17 (63.0)	43 (58.1)	0.699	12 (70.6)	25 (69.4)	0.876
**Anticholinergic treatment**		0 (0.0)	2 (2.7)	0.102	0 (0.0)	0 (0.0)	NA	1 (5.9)	1 (2.8)	0.266
**Monotherapy**		2 (7.4)	6 (8.1)	0.403	14 (51.9)	34 (45.9)	0.654	4 (23.5)	9 (25.0)	0.801
**Number of different antiparkinsonian drug types**		2.4 ± 0.9	2.8 ± 0.9	0.052	1.3 ± 0.7	1.6 ± 0.6	0.388	1.7 ± 0.7	1.9 ± 0.8	0.201
**Change in number of different antiparkinsonian drug types** ^**c**^		NA	NA	NA	−1.1 ± 0.8	−1.2 ± 0.9	0.556	−0.8 ± 0.8	−1.0 ± 0.8	0.321
**Number of different antiparkinsonian drug types**	**0**	0 (0.0)	0 (0.0)	0.189	0 (0.0)	0 (0.0)	0.201	1 (5.8)	1 (2.8)	0.301
	**1**	2 (7.4)	6 (8.1)		13 (48.1)	34 (45.9)		5 (29.4)	9 (25.0)	
	**2**	16 (59.3)	28 (37.8)		13 (48.1)	33 (44.6)		8 (47.1)	19 (52.8)	
	**3**	5 (18.5)	27 (36.5)		1 (3.7)	7 (9.5)		2 (11.7)	5 (13.9)	
	**4**	3 (11.1)	8 (10.8)		0 (0.0)	0 (0.0)		1 (5.8)	2 (5.6)	
	**5**	1 (3.7)	5 (6.8)		0 (0.0)	0 (0.0)		0 (0.0)	0 (0.0)	
**Number of tablets (pieces/day)**		7.4 ± 2.4	7.5 ± 2.3	0.678	4.3 ± 2.2	4.5 ± 2.5	0.433	4.8 ± 2.9	5.6 ± 2.7	0.542
**Change in number of tablets (pieces/day)** ^**c**^		NA	NA	NA	−3.1 ± 2.4	−3.0 ± 2.7	0.789	−2.6 ± 2.9	−1.9 ± 2.5	0.182
**LED for drugs containing levodopa (mg)**		857.3 ± 377.0	858.3 ± 361.1	0.806	373.2 ± 399.2	384.8 ± 414.4	0.366	411.5 ± 345.8	401.1 ± 370.5	0.433
**Change in LED for drugs containing levodopa (mg)** ^**c**^		NA	NA	NA	−482.1 ± 321.9	−497.1 ± 335.3	0.398	−442.7 ± 366.9	−455.8 ± 399.8	0.659
**LED for dopamine agonists (mg)**		314.3 ± 242.0	293.8 ± 215.2	0.628	271.2 ± 275.4	248.4 ± 265.5	0.750	235.5 ± 239.8	212.9 ± 206.7	0.444
**Change in LED for dopamine agonists (mg)** ^**c**^		NA	NA	NA	−44.2 ± 278.3	−49.6 ± 258.9	0.611	−69.3 ± 190.5	−55.4 ± 210.8	0.103
**Levodopa equivalent daily dose (mg)**		1212.9 ± 443.0	1265.9 ± 438.6	0.427	649.3 ± 438.2	650.1 ± 461.6	0.883	665.1 ± 384.1	671.6 ± 391.3	0.876
**Change in levodopa equivalent daily dose (mg)** ^**c**^		NA	NA	NA	−566.6 ± 452.1	−612.9 ± 443.6	0.665	−540.3 ± 399.1	−565.0 ± 405.4	0.511

Data are mean ± standard deviation or count (%).

Patient subgroups are the followings: Subgroup 1 = patients living close to the center and having the desired in-person visit number; while Subgroup 3 = patients living far from the center and having the desired in-person visit number.

^a^Based on data of 27 patients in subgroup 1 and 74 subjects in subgroup 3.

^b^Based on data of 17 patients in subgroup 1 and 36 subjects in subgroup 3.

^c^Change from preoperative status to first-year and five-year status.

^d^Preoperatively, MDS-UPDRS Part III was assessed in medication ON state, while it was assessed in medication ON and stimulation ON state at follow-up visits.

COMT = Catechol-O-methyl transferase inhibitor; LED = levodopa equivalent dose; MDS-UPDRS = Movement Disorder Society-sponsored Unified Parkinson’s Disease Rating Scale; NA = not applicable; NMDA = N-methyl-D- aspartate.

## Discussion

Remote DBS programming is an emerging innovation that seems to have several clinical and economic benefits^4–14^. In this study, we aimed to explore potential future beneficial effects of teleprogramming on the efficacy of stimulation treatment by analyzing the effects of travel distance and number of in-person visits on the one-year and five-year outcomes of DBS in PD.

According to our previous and present results, travel distance has a negative impact on the number of in-person follow-up visits both on short- and particularly long-term^12^, while lower number of in-person visits seems to be associated with a less favorable outcome of DBS especially on long-term. We found that PD patients who are living further than 50 km from our center and had less in-person follow-up visits than considered as optimal by our protocol before the introduction of remote DBS programming showed worse HRQoL and symptomatic improvement at the five-year follow-up compared to those far-living subjects who had regular in-person visits, while after one year of stimulation the outcome of these two groups of patients was almost comparable. In the analyzed groups, there was a difference in the numbers of in-person visits already in the first year, however, this difference became more pronounced over the remaining 4 years of the analyzed follow-up period. Long-distance travels seem to be one of the underlying causes for the decreasing number of follow-up visits among a not negligible part of DBS patients who live far from the movement disorder center (42% in the present study) over the follow-up period after the implantation. However, before the availability of remote DBS programming, travel for in-person visits was a prerequisite for optimalization of stimulation treatment which put high financial burdens on and required time from patients and their caregivers^12^. Patients who live closer to the center may be more likely to visit their doctors in-person to discuss any problems and before remote programming, this provided the opportunity to clinicians for checking and optimizing the stimulation. Patients who live far from the center may be more likely to contact the center on other routes (e.g., via phone and e-mail), however, without remote programming, this does not allow clinicians to optimize DBS settings. If the patient cannot attend the necessary follow-up visits for optimizing the parameters of the stimulation among others because of mobility disturbances, worsening disability resulting from both suboptimal stimulation and disease progression can become a barrier to travel^12^. The more severe the mobility problems, the harder it is for the patient to travel long distances. Without remote programming, if the patient cannot travel to the center, the stimulation cannot be optimized. Based on this, an assumed bidirectional relationship between travels for in-person visits and regular optimalization of DBS settings may be a factor to be considered in far-living PD patients treated with DBS.

At the one-year follow-up visit, compared to the preoperative status, DBS led to clinically important improvement in HRQoL in both analyzed groups according to the PDQ-39 SI, however, at five years, this improvement was significantly larger and remained clinically relevant only in those far-living patients who had the desired number of in-person visits over five years^15^. In this group of patients, the improvement in the EQ-5D-5 L summary index was also significantly larger both at the first-year and five-year follow-up visits, and considering the lowest reported minimal clinically important difference (MCID) thresholds for improvement in the summary index of the EQ-5D (from 0.03 to 0.08)^16–18^ and the MCID threshold for the Hungarian population (0.0705)^19^, only the improvement measured in far-living patients with regular follow-up visits exceeded these values at five years. Larger improvement in mobility and ability to perform activities of daily living (ADL) were the main contributors to the better HRQoL in the group of patients who visited our unit at least as frequently as considered as optimal. A study by Gorecka-Mazur et al. found significant correlation between ADL performance and HRQoL after subthalamic DBS, and this study also showed that DBS has the largest improving effect on the HRQoL in the first 3–6 months of stimulation which improvement remains stable during the remaining follow-up period^20^. These results, in line with some other findings^21–23^, highlight the importance of regular visits and on-demand optimalization of stimulation settings in the early follow-up period. In our study, the difference in number of in-person visits was smaller and there was no significant difference in global HRQoL and ADL performance between the compared far-living patient groups during the first year, however, over five years, difference in number of in-person visits increased between the far-living groups and beneficial effects of DBS on HRQoL and ADL could be maintained only in patients with regular follow-up visits. In addition, our results did not show difference in HRQoL measures between patients who live close to our center and had the desired number of in-person visits and those who live far from our unit and visited our center regularly either during short- or long-term follow-up. Based on this, regular follow-up visits and on-demand optimalization of stimulation parameters seems to be important also in maintaining the effectiveness of DBS^21–23^. Continuously maintaining an effective treatment is of high importance in several aspects of everyday living of a PD patient because among others losing ability to work seems to have irreversible negative impact on beneficial effects of DBS on the HRQoL^24^.

In our study, patients who visited our center regularly despite long-distance travel showed similar motor and non-motor symptomatic control than patients who had to travel shorter distances to visit our unit both during short- and long-term follow up. Of the motor symptoms, disabling dyskinesia including OFF periods with painful dystonia and choreiform hyperkinesia is one of the main indications for DBS in PD. Depending on the target of stimulation, DBS can directly or by enabling dose reduction in oral antiparkinsonian treatment, indirectly decrease the severity of dyskinesia and the impact of dyskinesia on ADL^25,26^. In our study, dyskinesia could be reduced in a clinically relevant manner^27^ in both far-living groups of patients at the first-year follow-up visit, however, this beneficial effect of DBS remained clinically meaningful only in those far-living patients who visited our center regularly. Higher number of patients on levodopa treatment which is a main contributor to the development of dyskinesia in PD and presumably suboptimal DBS settings in the group of far-living patients who did not have the desired number of in-person visits can partially explain the previous finding. In contrast, dopamine agonists could be used in a higher portion of far-living patients with regular in-person follow-up visits. Based on these results, it also seems that number of in-person visits and possibility of maintaining the optimal stimulation treatment may influence the choice of adjuvant antiparkinsonian medications which can have both clinical and economic consequences^28,29^. In addition to possible beneficial effect of remote programming on patient compliance to regular check-ups regarding stimulation treatment, possibility of greater simplification to per oral antiparkinsonian pharmacotherapy on long-term with regular DBS adjustments may have not just beneficial economic effects but increase the patient compliance also with pharmacological treatment. However, regarding this, it should be also noted that subtype and leading symptoms of PD may influence both adjustments to per oral antiparkinsonian medications postoperatively and DBS programming. As an example, tremor-dominant patients may need higher frequency stimulation to relive tremor, however, it has been described that high frequency stimulation may worsen gait difficulties and postural instability which is not optimal therefore for patients having such problems^30^. Tremor may poorly be responsive to levodopa which may necessitate the use of other antiparkinsonian medications such as dopamine agonists and anticholinergics^31^, however, the latter may again worsen gait difficulties and postural instability^32^. In addition to subtype of PD and clinical symptoms, stimulation parameters may also have an impact on per oral antiparkinsonian treatment. In our study population, distribution of PD subtypes was balanced across the subgroups.

Although this analysis is based on data collected by only our center, because our movement disorder unit is a tertiary center, we could include a relatively large number of patients from the whole area of the country. However, during the interpretation of our results, some limitations should also be considered. This study was a retrospective analysis of data from our prospective movement disorder registry and from healthcare information systems, and neither our registry nor the other used systems contain data on the exact cause of not attending our unit. Therefore, the present analysis could not exactly reveal all possible causes of and the level of their contributions to not achieving the optimal number of in-person visits. We found a negative correlation between travel distance and the number of in-person visits, and assumed that mobility disturbances and worsening disability resulting from both suboptimal stimulation in the lack of the desired number of in-person visits providing the opportunity for optimizing the stimulation and disease progression can become a barrier to travel. It can be suspected that long-distance, time-consuming and costly travel can be one of the main contributors to not attending the center among patients living far from the center. However, some other factors such as unsatisfactory outcome after DBS implantation may also contribute to not achieving the desired number of visits but one can assume that factors less related or unrelated to travel are more important for patients living closer to the center. Remote programming may be a useful tool also for patients in whom it takes longer time to find the optimal settings but lose motivation for travelling long distances because not having the desired improvement after the first few programming sessions or experiencing decreasing efficacy as the disease progresses. During the interpretation of our results, it should also be considered that clinical practice on selecting antiparkinsonian medications after initiating the stimulation treatment and protocol for DBS care may vary among centers.

According to the results of a search in the PubMed database conducted on February 12, 2025 using the terms ‘deep brain stimulation’, ‘patient selection’, ‘decision making process’, ‘effectiveness’ and ‘cost-effectiveness’, in available articles from the era before the wider introduction of DBS teleprogramming, travel distance between the residence of the DBS candidate and the clinic has not been taken into account during the selection of patients for whom DBS may be effective and cost-effective. However, according to our results, travel distance might have been an important but overlooked factor influencing long-term DBS outcomes primarily through its impact on access to in-person follow-up and stimulation optimization. Travel distance would have been therefore a not negligible factor during selecting the best PD candidates for DBS treatment. By highly decreasing the impact of travel distance on stimulation treatment, remote programming may be a feasible tool to make close- and far-living PD patients equal candidates for DBS, however, analyses of real-life data on DBS teleprogramming should confirm this hypothesis.

## Materials and methods

### Ethical approval

All study-related procedures were approved by the National Ethical Board of Hungary and the National Institute of Pharmacy and Nutrition (031037/2015/OTIG and OGYÉI/6916/2021). The study was performed in accordance with the Declaration of Helsinki. Our registry partly serving as a source of data analyzed in this study fulfills all security and data protection requirements and was approved by the Hungarian Medical Research Council.

### Study population

From our MD registry, all subjects who fulfilled the following criteria were included: the patient (1) must have signed an informed consent form approved by the respective Ethical Committees before the inclusion in our MD registry; (2) underwent DBS implantation for medication-refractory PD; (3) had the DBS system activated at our clinic; and (4) had at least a one-year follow-up in our unit after the activation. Patients who had the one-year follow up after August 12, 2022 were excluded. Five-year outcome data of subjects who had the five-year follow up visit after August 12, 2022 was also not used. Our center started using the NeuroSphere™ platform by Abbott on August 12, 2022, and teleprogramming may have had an impact on the number of in-person visits and DBS outcome.

### Sources of data

This study was based on the prospective MD registry of our department which is a tertiary MD center in Hungary. It served as the basis for identifying patients eligible for inclusion in the analyses, and it also included all data on the one-year and five-year outcomes of the stimulation treatment.

Additional data (e.g., address of the patient, number of in-person visits during the first year and five years of stimulation) were collected from the healthcare information systems used at our unit. During the analyses, we considered only those in-person visits where changes to stimulation settings (e.g., changing the lower and/or upper limits of patient amplitude control, the contact used for stimulation, the frequency or pulse width of the stimulation or initiating a completely new program) were performed.

Travel distances and times between the homes of subjects and our department were determined using the route planer called ViaMichelin (https://www.viamichelin.com). In every case, the fastest route was considered. Hatchback and E5 unleaded petrol were selected for type of car and type of fuel, respectively.

We used data of patients who signed an informed consent form prior to all study related procedures.

### Primary endpoint

Our main aim was to explore the effects of travel distance and number of in-person visits during the first year and five years of stimulation treatment on HRQoL measured by different dimensions and the summary index of the PDQ-39 and the generic quality of life scale called EQ-5D-5 L at the one-year and five-year follow-up visits. To measure the level of independence in daily chores, an important contributor to HRQoL, the SE-ADL was also assessed both at the one-year and five-year follow-up.

### Secondary endpoint

We also investigated the effects of travel distance and number of in-person visits during the first year and five years of DBS treatment on the motor and non-motor outcome of DBS measured by different parts of the MDS-UPDRS at the one-year and five-year follow-up visits.

### Patient categorization

Patient subgroups were developed based on travel distance between the home of the patient and our center and the number of in-person-visits during the first year and five years of stimulation treatment. Because about 50 km can be generally travelled over 60 min in Hungary and our unit can also be reached within 60 min from most parts of Baranya county in which Pécs lies, one of the grouping aspects was whether the patient lives close (≤ 50 km) to or far (> 50 km) from our center. Included subjects were further categorized based on the number of in person visits during the first year and five years. Our protocol considered as optimal if the patient had at least 3 visits (3 months, 6 months, and 12 months after activation) during the first year of DBS treatment and at least 2 in-person visits per year from the second year which means that a patient who was being treated with DBS for PD should have had at least 11 visits over a 5-year follow-up period. Based on these aspects, the following groups of patients were developed for both the first-year and the five-year analyses: (1) living close to the center and having the desired in-person visit number, (2) living close to the center and having less in-person visits than desired, (3) living far from the center and having the desired in-person visit number, and (4) living far from the center and having less in-person visits than desired.

### Statistical analysis

Because most of our PD patients treated with DBS live further than 50 km from our center, the large difference in the numbers of subjects living close and far did not allow us to reliably compare data of these two groups of patients. Therefore, we decided to compare DBS outcomes of far-living patients who had and missed the desired numbers of in-person visits for the first year and five years of stimulation treatment. To test normality, the Kolmogorov-Smirnov test or the Shapiro-Wilk test was used depending on sample size. Because data did not follow the normal distribution, to analyze correlation between travel distance and numbers of in-person visits during the first year and five years, Spearman’s rank correlations were used. To compare data of patient subgroups, Mann-Whitney U and Chi-squared tests were performed. The level of statistical significance was set at 0.05. All statistical analyses were performed using jamovi version 2.2.5.

## Data Availability

The data used to support the findings of this study have not been made publicly available because the ethical approval does not permit their deposition. They are available from the corresponding author only on reasonable request.

## Abbreviations

ADL: Activities of daily living
CGI-I: Clinician Global Impression of Improvement Scale
DBS: Deep brain stimulation
EQ-5D-5 L: EuroQoL 5-Dimension 5-Level
HRQoL: Health-related quality of life
MD: Movement disorder
MDS-UPDRS: Movement Disorder Society-sponsored Unified Parkinson’s Disease Rating Scale
MCID: Minimal clinically important difference
PD: Parkinson’s disease
PDQ-39: 39-item Parkinson’s Disease Questionnaire
SE-ADL: Schwab-England Activities of Daily Living Scale
